# Supplementary material for: Causal Relationship between Aging and Anorexia Nervosa: A White-Matter-Microstructure-Mediated Mendelian Randomization Analysis
Source: Biomedicines. 2024 Aug 16;12(8):1874. doi: 10.3390/biomedicines12081874 (PMC11351342; doi:10.3390/biomedicines12081874)
Supplement: Supplementary file 1 [file biomedicines-12-01874-s001.zip › Supplementary Figure/Figure S8/Leaveoneout plot of MR analysis for TL to ubm-b-1475.pdf]

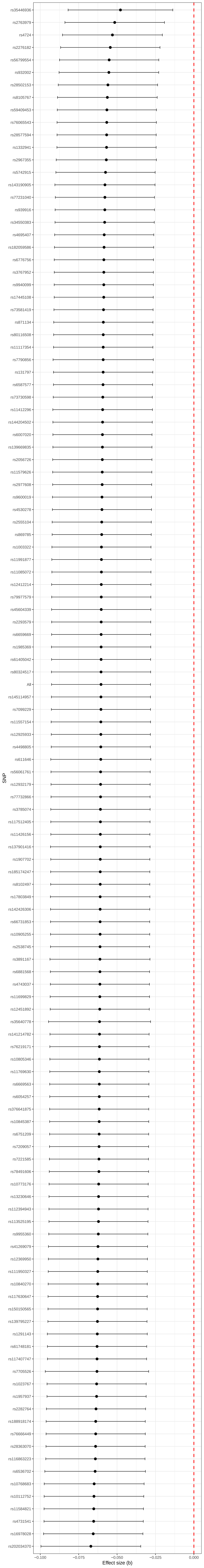

SNP
Effect size (b)
-0.100
-0.075
-0.050
-0.025
0.000
rs35446936
rs2763979
rs4724
rs2276182
rs56799554
rs932002
rs28502153
rs8105767
rs59409453
rs76065543
rs28577594
rs1332941
rs2967355
rs5742915
rs143190905
rs77231040
rs939916
rs34550383
rs4695407
rs182059586
rs6776756
rs3767952
rs9940099
rs17445108
rs73581419
rs871134
rs80116508
rs11117354
rs7790856
rs131797
rs6587577
rs73730598
rs11412296
rs144204502
rs6007020
rs139669835
rs2056726
rs11579626
rs2977608
rs9600019
rs4530278
rs2555104
rs869785
rs1003322
rs11991877
rs11085072
rs12412214
rs79977579
rs45604339
rs2293579
rs6659669
rs1985369
rs61405042
rs80324517
All
rs145114957
rs7099229
rs11557154
rs12925933
rs4498805
rs611646
rs56061761
rs12932179
rs77732866
rs3785074
rs117512405
rs11426156
rs137901416
rs1907702
rs185174247
rs8102497
rs17803849
rs142426306
rs66731853
rs10905255
rs2538745
rs3891167
rs6881568
rs4743037
rs11699829
rs12451892
rs35640778
rs141214782
rs76219171
rs10805346
rs11769630
rs6669563
rs6054257
rs376641875
rs10845387
rs6751209
rs7209057
rs7221585
rs78491606
rs10773176
rs13230646
rs112394943
rs113525195
rs9955360
rs41269079
rs12369950
rs111950327
rs10840270
rs117630647
rs150150565
rs139795227
rs1291143
rs61748181
rs117407747
rs7705526
rs1023767
rs1957937
rs2282764
rs188918174
rs76666449
rs28363070
rs116863223
rs6536702
rs10768683
rs10112752
rs11584821
rs4731541
rs16978028
rs202034370
